# Cardiovascular Complications from Tuberculosis

**DOI:** 10.31083/RCM44092

**Published:** 2025-11-25

**Authors:** Germantė Mikalajūnaitė, Egidija Rinkūnienė, Alma Čypienė, Vilma Dženkevičiūtė, Jolita Badarienė

**Affiliations:** ^1^Institute of Clinical Medicine, Clinic of Cardiac and Vascular Diseases, Faculty of Medicine, Vilnius University, 03101 Vilnius, Lithuania; ^2^Clinic of Cardiac and Vascular Diseases, Vilnius University Hospital Santaros Klinikos, 08406 Vilnius, Lithuania; ^3^Center of Cardiology and Angiology, Vilnius University Hospital Santaros Klinikos, 08406 Vilnius, Lithuania

**Keywords:** tuberculosis, cardiovascular complications, pericarditis, myocarditis, aortitis

## Abstract

Tuberculosis (TB) is a contagious disease caused by *Mycobacterium tuberculosis *(*M. tuberculosis*) and is transmitted through airborne particles. Although TB usually damages the lungs, this disease can also cause complications in various organs, including the cardiovascular system. Indeed, pericarditis represents the most frequently reported cardiac manifestation of TB, and may present alongside fever, dyspnea, cough, or increased central venous pressure, hepatomegaly, and peripheral edema. Tuberculous-related pericarditis treatment is challenging due to the poor penetration of anti-tuberculous drugs into the pericardium. Myocarditis is another form of cardiac manifestation and is often associated with arrhythmias. Tuberculous aortitis typically causes dilatation leading to pseudoaneurysm formation and is usually asymptomatic; however, this manifestation can result in sepsis, aortic rupture, or even death, although rarely. Cardiac tuberculomas may present with general symptoms and can impair heart function by obstructing the outflow tracts, leading to ventricular dysfunction. Additionally, the primary treatment of TB carries cardiotoxicity risks, such as various arrhythmias. Moreover, TB significantly increases the risk of cardiovascular conditions, including myocardial infarction and coronary artery obstruction. Therefore, early recognition and a multidisciplinary approach are crucial to prevent severe outcomes such as sudden cardiac death, sepsis, or aortic rupture. Thus, this review highlights the spectrum of TB-related cardiac complications and underscores the importance of greater awareness and timely multidisciplinary care.

## 1. Introduction

Tuberculosis (TB) is an airborne infectious disease caused by *Mycobacterium Tuberculosis* (*M. tuberculosis*). It predominantly affects the lungs, but can disseminate throughout the body and damage other organ systems, such as the gastrointestinal, central nervous system, or cardiovascular [1]. According to the recent World Health Organization (WHO) Global Tuberculosis Report 2024, the global TB incidence rate (new cases per 100,000 population per year) increased by 4.6% between 2020 and 2023, with a slowdown in the last year; however, between 2010 and 2020, an annual 2% decrease was observed. Also, TB has potentially regained as the leading global cause of mortality from a single infectious agent, replacing COVID-19 [2]. Most TB cases were registered in South-East Asia (45%), Africa (24%) and the Western Pacific (17%) regions, which represent countries with low socioeconomic status (low income, limited education, crowdedness and lack of employment)—one of the main TB risk factors, as well as human immunodeficiency virus (HIV), immunosuppressive treatment, chronic illnesses (diabetes, rheumatoid arthritis and other), malnutrition, alcohol or tobacco use [2, 3].

Cardiovascular involvement in tuberculosis primarily affects the pericardium, less often the myocardium, and the aorta [4]. Even though the disease is completely curable, in 2023 there were 1.25 million deaths due to TB, with a global mortality rate was 2.4 per 100,000 population. For instance, tuberculous pericarditis carries a 40% risk of death at 6 months in patients with HIV infection and 25% in those without HIV [2, 5]. To prevent potentially serious cardiac complications in tuberculosis, attentive cardiological examination and tracking are recommended [4, 6].

The main aim of this literature review is to provide a comprehensive summary of recent scientific literature on cardiac involvement in tuberculosis, epidemiology, clinical presentation, and potential outcomes.

## 2. Literature Review

### 2.1 Tuberculous Pericarditis

TB is considered the leading cause of pericardial diseases worldwide, particularly in low and middle-income countries [7, 8]. Among individuals with pulmonary TB, approximately 1–2% develop TB pericarditis, which is associated with a substantial 6-month mortality ranging from 17% to 40%. If left untreated, up to half of cases progress to cardiac tamponade, with mortality rates reaching 85% at 6 months [9, 10]. However, global data on the incidence and outcomes of TB pericarditis are limited, as most available studies originate from endemic regions. *M. tuberculosis* mostly reaches the pericardium by spreading through the lymphatic system, though in HIV patients, hematogenous spread is the most common route, and in rare cases, there is a direct contiguous spread from nearby structures such as the lungs, pleura, or spine [10, 11]. Although TB primarily affects the lungs, rarely, but there are some clinical cases that report TB pericarditis as the only manifestation [12, 13, 14].

#### 2.1.1 Pathophysiology

The clinical presentation of TB pericarditis can manifest in four different stages: dry, effusive, adsorptive, and constrictive (Table 1). In the dry stage, the entire pathological process begins in the pericardium when *M. tuberculosis* antigens are presented by macrophages to CD4+ T lymphocytes, triggering the activation of lymphocytes, macrophages, and complement-fixing antibodies. It results in the inflammation of pericardial leaflets, granuloma formation, cytolysis, and a fibrous exudate with an abundance of inflammatory cytokines. Notably, HIV patients have low CD4+ T cell levels, which leads to low immune response, and this process is disrupted [9, 15]. The dry stage is the least common, but it presents with symptoms of acute pericarditis and ST-segment elevation in the electrocardiogram (ECG). It has been observed that the predominant acute TB pericarditis symptoms are fever (73%) and cough, dyspnea, chest pain (41–46%), while stabbing pain and pericardial rubbing, which are typical symptoms of acute pericarditis, are uncommon in TB pericarditis [10]. Another study in an intermediate-burden country noticed that the most frequently observed symptom was dyspnea (84.1%) and is associated with a higher risk of unfavourable outcomes such as cardiac tamponade, constrictive pericarditis, and death [16].

**Table 1.** **Summary of all TB pericarditis stages**.

Stage	1. Dry	2. Effusive	3. Adsorptive	4. Constrictive
Percentage	1–2%	79.5%	Rare	5–25%
Pathophysiology	Fibrinous exudation, early granuloma formation with macrophages and T cells	Serosanguineous effusion	Effusion absorption, organized granulomatous caseation, pericardial fibrous thickening, fibrosis	Constrictive pericardial scarring and/or calcification
Pericardial fluid	Low polymorphonuclear, predominantly Mtb concentration	Predominantly lymphocytic exudate	Pericardial fibrous thickening -fibrin deposition and collagen accumulation	No residual fluid in the pericardium
Clinical signs and symptoms	Mostly asymptomatic	Fever, cough, dyspnea, chest pain	Increased central venous pressure, hepatomegaly, peripheral edema, ascites, muffled heart sounds, sinus tachycardia, palpable apical impulse	
Imaging findings	Widespread ST elevation, PR depression, non-specific T wave changes in the electrocardiogram (ECG)		Thick fibrinous fluid around the heart	Little or no pericardial effusion
Chest x-ray	Enlargement of cardiac silhouette		Pericardial layer calcifications	
Echocardiography	∙ Echo-free space between the two layers of the pericardium (global or localised):		∙ Respirophasic ventricular septal shift (also called septal bounce)	
	∘ Mild effusion: <10 mm separation between pericardial layers		∙ Increased mitral E-wave velocity and E/A ratio >1.6 (in expiration)	
	∘ Moderate: 10–20 mm separation		∙ Respiratory variation of peak mitral E-wave velocity (at least >15%)	
	∘ Severe: >20 mm separation		∙ Prominent expiratory diastolic flow reversal in hepatic veins	
	∙ Fibrinous strands and clots (chronic)		∙ Preserved or exaggerated medial mitral annulus early diastolic (e’) velocity (≥9 cm/s)	
	∙ Diastolic collapse of cardiac chambers (cardiac tamponade)		∙ Medial e’ equal to or greater than lateral mitral annulus e’ velocity (annulus reversus)	
	∙ Diastolic ventricular variability with respiratory cycle size (cardiac tamponade)		∙ Constrained circumferential and preserved longitudinal myocardial deformation (strain)	
	∙ Inferior vena cava (IVC) dilation (cardiac tamponade)			
	∙ Septal bounce (cardiac tamponade)			

TB, tuberculosis; ST, ST-segment; PR, PR interval; ECG, electrocardiogram.

In some patients, dry pericarditis may progress to a second stage—effusive pericarditis, which is the most common TB pericarditis presentation: a systematic review of 36 studies on pericardial cases in Africa, an endemic region of TB, revealed that 79.5% of tuberculous pericarditis cases presented as effusive pericarditis [5]. It is the large accumulation of fluid in the pericardium that compresses the heart chambers, affecting cardiac filling and contraction. At this stage, pericardial fluid contains a lymphocytic exudate with monocytes and foam cells, and patients may present with signs and symptoms of heart failure. If fluid accumulation in the pericardium occurs suddenly and rapidly, the patient may present with tachycardia, hypotension, and/or evolve into tamponade (20%). Moreover, in this stage, the combination of effusion and constriction may occur when the pericardium constricts and pericardial fluid simultaneously accumulates [4, 9, 10]. In this stage, changes in the pericardium may be detected using imaging techniques, with echocardiography recommended as the first-line investigation. Pericardial effusion may appear as an echo-free (anechoic or hypoechoic) space between the separated layers of the pericardium. Moreover, in cases of large or chronic effusion, fibrinous strands may be visible [4, 17].

In the absorptive stage, the pericardial effusion begins to be absorbed, a granulomatous caseation starts to organise, and a pericardial fibrous thickening occurs because of fibrin deposition and collagen accumulation. Echocardiographically, it may appear as a thick fibrinous fluid surrounding the heart [9].

When no pericardial fluid remains, it is referred to as the constrictive stage, which is associated with the poorest prognosis regarding associated dysfunction. This stage is characterized by a loss of pericardial elasticity and calcification, with little or no effusion, resulting in reduced cardiac diastolic filling [4, 9]. It presents as heart failure symptoms with progressive fluid retention: increased central venous pressure (100%), hepatomegaly (100%), peripheral edema (94%), ascites (89%) and less common symptoms such as muffled heart sounds (76%), sinus tachycardia (70%) and palpable apical impulse (58%) [10]. The main echocardiographic signs of constrictive pericarditis are no residual fluid in the pericardium, respirophasic ventricular septal shift (septal bounce), increasing mitral E-wave velocity and E/A ratio >1.6 (in expiration), constrained circumferential and preserved longitudinal myocardial deformation (strain), and other (Table 1) [18]. Cardiac computed tomography (CT) is the gold standard for imaging pericardial calcifications, pericardial thickening >3–4 mm characteristic of constrictive pericarditis may also be visible [4]. A summary of all TB pericarditis stages and their main findings is described in Table 1 [9, 17, 18, 19].

One Investigation of the Management of Pericarditis (IMPI) trial analyzed 1370 adult patients (median age for HIV-positive individuals is 34.0 years, for HIV-negative individuals 47.7) with TB pericarditis, of whom 939 were co-infected with HIV. The results showed that HIV-infected patients were approximately 13–14 years younger than those without HIV. They had a higher occurrence of tachycardia, hypotension, anemia, and radiographic pulmonary infiltrates indicating TB, but had a lower incidence of peripheral edema. Furthermore, it was noted that HIV infection modifies the cardiovascular presentation and reduces the incidence of constrictive pericarditis, without increasing the mortality rate [20]. These changes can be explained by immunological mechanisms, as demonstrated in a study that analyzed pericardial fluid contamination using the flow cytometric method. It showed that in HIV-positive patients, CD4+ memory cells exhibit a less differentiated phenotype with reduced expression of tumor necrosis factor (TNF), interleukin-2 (IL-2), and interferon-gamma (IFN-γ), which are significant cytokines in the immune response. It results in an increased risk of developing TB pericarditis [21]. The main differences between TB pericarditis in HIV-negative and HIV-positive patients are described in Table 2 [9, 20].

**Table 2.** **Differences between TB pericarditis in HIV-negative and HIV-positive patients**.

	HIV-negative	HIV-positive
Age	About 34 years old	About 47–48 years old
Common mechanism of TB spread	Lymphatic system	Hematogenous dissemination
Common symptoms	Fever, cough, dyspnea, chest pain, night sweats, weight loss	
	Peripheral edema	Tachycardia, hypotension, anemia
Pericardial fluid (PF)	Dominates CD8+ T cells	Dominates CD4+ T cells
	Less viral load than in plasma (Viral load: PF < plasma)	Higher viral load than in plasma (Viral load: PF > plasma)
Prognosis	Acute TB pericarditis – poor prognosis (17–40% die in 6 months)	Poor prognosis
		Less chance of developing constrictive pericarditis
		Treatment linked to increased risk of malignancy

HIV, human immunodeficiency virus.

#### 2.1.2 Diagnosis

Diagnosing TB pericarditis is a difficult challenge for healthcare workers. It is estimated that 15–20% of all pericardial diseases remain undiagnosed and have an elevated risk of morbidity and mortality [9]. Imaging tests play a key role in diagnosing TB pericarditis. Echocardiography is the most used imaging modality, helping to identify pericardial fluid, which appears as an area of increased echogenicity due to a large amount of fibrin or fibrous strands, and to assess its volume. Moreover, in chest radiography, effusive TB pericarditis may present as an enlarged cardiac silhouette, while in the constrictive stage, pericardial layer calcifications may be observed. The most diagnostically informative imaging test is a chest CT scan, which can provide detailed information about pericardial fluid volume, localization, density, thickness, and/or calcifications. Enlarged mediastinal and tracheobronchial lymph nodes (>10 mm), which may be present in patients with TB pericarditis, can also be observed [5]. Fluorodeoxyglucose positron emission tomography ([^18^F]-FDG-PET/CT) is a modern and especially valuable imaging modality, as it provides the most accurate diagnosis of TB pericarditis, lymph node involvement, selection of the optimal biopsy site, and assessment of treatment efficacy [22]. Magnetic resonance imaging (MRI) can also help distinguish a small amount of pericardial fluid from pericardial thickening. Additionally, it helps to identify constrictive pericarditis as it shows the real-time dynamic (systole and diastole) assessment of cardiac function: it visualizes the movements of the ventricular septum and monitors ventricular filling during inspiration. Although it offers poorer visualization of calcifications and lung parenchyma [5]. According to the 2015 European Society of Cardiology Guidelines for pericardial diseases, for patients with a minimal amount of pericardial fluid volume (<10 mm) and suspected tuberculous pericarditis, the diagnostic approach should begin with investigating tuberculosis in other locations, for example, by performing bronchoscopy with bronchial secretion sampling for microbiological and genetic testing or by taking a biopsy of enlarged lymph nodes. Moreover, urgent pericardiocentesis must be done in patients who have developed cardiac tamponade or rapidly progressive, hemodynamically significant pericardial effusion. In cases of recurrent cardiac tamponade, surgical placement of the pericardial drain is recommended [7].

The key diagnostic markers of TB pericarditis in a sterile pericardial sample include *M. Tuberculosis* cultures found with acid-fast bacilli staining, polymerase chain reaction (PCR) for *M. Tuberculosis* DNA, along with adenosine deaminase (ADA) >40 U/L, which shows a lymphocyte-predominant effusion, and unstimulated IFN-γ, which is a cytokine secreted by Th1 cells during the immune response [7, 23]. If there are no feasible methods to obtain pericardial fluid in endemic regions, a diagnosis of TB pericarditis can be established using a diagnostic score based on the following criteria: fever (1 point), night sweats (1 point), weight loss (2 points), globulin level >40 g/L (3 points), and peripheral leukocyte count <10 × 10^9^/L (3 points). A score of ≥6 strongly suggests TB pericarditis (Table 3) [7].

**Table 3.** **Diagnostic criteria for TB pericarditis in endemic areas**.

Criteria	Points
Fever	1
Night sweats	1
Weight loss	2
Globulin level >40 g/L	3
Peripheral leukocyte count <10 × 10^9^/L	3
A total score ≥6 highly suggests TB pericarditis.	

Constrictive pericarditis must be differentiated from restrictive cardiomyopathy, as the two conditions present with similar features. The key factors supporting the diagnosis of constrictive pericarditis include echocardiographic findings such as septal bounce, pericardial thickening and/or calcifications, respiratory variations of the mitral inflow (peak E velocity >25%, pulmonary venous peak D flow velocity >20%), tissue Doppler peak e’ >8 cm/s, while in cases of restrictive cardiomyopathy there is a small left ventricle and large left atrium, there is no significant changes in mitral inflow during respiration, and tissue Doppler peak e’ <8 cm/s [4].

#### 2.1.3 Treatment

The prognosis of patients with acute TB pericarditis remains poor (17–40% of patients diagnosed with TB pericarditis die in 6 months) despite adequate four anti-TB drugs treatment (isoniazid, rifamycin, pyrazinamide, and ethambutol) due to insufficient penetration from plasma to pericardial fluid. A pilot study analyzed 16 patients treated for TB pericarditis, focusing on measurements of anti-TB drugs, pH, and protein in pericardial TB fluid compared to plasma. The results showed that free (non-protein-bound) rifampicin concentration in pericardial fluid in no sample exceeded the minimum inhibitory concentration (MIC) with a value of 0.208 mg/L, while the highest detected concentration was 0.125 mg/L (*p* = 0.001). The free peak concentration of ethambutol (*p* < 0.001) and pyrazinamide (*p* < 0.0001) were also below the MIC, whereas only isoniazid exceeded the MIC and penetrated to adequate concentrations [19, 24]. Although additional steroid therapy does not affect mortality, it is associated with decreased hospitalization, progression of TB effusive pericarditis to constrictive pericarditis, and a reduced need for recurrent pericardiocentesis. However, in HIV-positive individuals, it is linked to an increased risk of malignancy [25, 26].

### 2.2 Tuberculous Myocarditis

TB myocarditis is a very rare extrapulmonary TB manifestation and is most commonly diagnosed only postmortem during autopsy [27, 28]. As well as TB pericarditis, *M. Tuberculosis* can reach the myocardium through hematogenous or lymphatic spread, or even directly. However, some literature suggests that the most common is the hematogenous pathway [10, 29]. One study noted that TB myocarditis is usually diagnosed in patients with a normal immune system (not HIV-positive), and in most patients under 45 years old, it is the sole sign of TB [29].

Some literature suggests that there is a higher risk of TB myocarditis on the right side of the heart due to a tendency to primarily involve the right-sided mediastinal lymph nodes, while one systematic review found that 68% of all cases of TB mainly involved in left ventricle, less common myocarditis damaged the right ventricle and atrium, and at least – left atrium. It is important to acknowledge that conflicting results may be attributable to limited data and potential bias [10, 28, 29]. In autopsy, it may present as nodular tuberculomas with central caseation, miliary tubercles in the heart, or a diffuse infiltrative pattern linked to pericarditis [28].

Clinically, TB myocarditis may present as the only TB manifestation, together with pericarditis as myopericarditis, and in rare cases, can be asymptomatic or with different heart conduction system disorders [29, 30]. Myocarditis in the ECG may show up as long QT interval syndrome, p pulmonale, right bundle branch block, or other nonspecific ventricular arrhythmias, for example, ventricular tachycardia [27, 31, 32]. Moreover, it can cause chest pain, dyspnea, heart failure, lymphadenopathy, or even sudden cardiac death [31, 32, 33]. It is essential to note that these symptoms are non-specific and may mislead healthcare specialists, as acute TB myocarditis can be misinterpreted as acute myocardial infarction or chronic myocarditis may present with symptoms similar to chronic heart failure [33].

Severe laboratory findings may help in the diagnosis of TB myocarditis, including elevated troponin and C-reactive protein (CRP) levels. Echocardiographic abnormalities may include regional wall motion or biventricular (global) dysfunction. On cardiac magnetic resonance (CMR) imaging, TB myocarditis may present as myocardial edema, inflammation, or fibrosis [31, 33].

It should be emphasized that TB myocarditis usually responds to anti-TB therapy, but the risk of sudden cardiac death remains unchanged despite the treatment. Therefore, continuous patient monitoring and intensive management of symptoms are crucial [29].

Table 4 summarizes and compares the diagnostic findings in TB pericarditis and TB myocarditis based on [5, 7, 23, 27, 31, 32, 33]. In summary, TB pericarditis may clinically present with fever and/or cough, as well as peripheral edema. Echocardiographically, the space between the two pericardial layers may be observed, and most importantly, pericardial fluid laboratory analysis may be useful, revealing the presence of *M. tuberculosis* bacilli along with elevated adenosine deaminase and interferon-gamma levels. In contrast, TB myocarditis may present with new-onset chest pain, dyspnea, and heart failure, accompanied by distinct ECG abnormalities, and echocardiography may reveal new myocardial dysfunctions, while no pericardial effusion may be present.

**Table 4.** **Comparison of diagnostic findings in TB pericarditis and TB myocarditis**.

	TB pericarditis	TB myocarditis
Most common reach	By the lymphatic system	By hematogenous spread
Symptoms	∙ **Fever, cough, dyspnea**, chest pain (effusive stage)	∙ **Chest pain, dyspnea, heart failure**, lymphadenopathy
	∙ **Peripheral edema, ascites**, sinus tachycardia (constrictive stage)	
ECG	PR depression, ST elevation	Long QT syndrome, p pulmonale, right bundle branch block, unspecific ventricular arrhythmias
Echocardiography	Echo-free (anechoic or hypoechoic) **space between the separated two layers of the pericardium** (increased pericardial fluid volume), fibrin or fibrous strands, septal bounce, and pericardial thickening	Newly developed regional wall **motion abnormalities** or global **ventricular dysfunction**
MRI/CMR	Ventricular interdependence on real-time cine MRI	Myocardial edema, inflammation, or fibrosis
CT	Pericardial thickness >3–4 mm, pericardial calcifications	Similar to MRI/CMR
Pericardial fluid	∙ ***M. tuberculosis* bacilli**	Without changes, except if the pericardium is involved (myopericarditis)
	∙ ↑**Adenosine deaminase (ADA) >40 U/L**	
	∙ ↑**Interferon-gamma (IFN-γ)**	

Bold font indicates the most important findings. MRI, magnetic resonance imaging; CMR, cardiac magnetic resonance; CT, computed tomography; *M. tuberculosis*, *Mycobacterium tuberculosis*; ↑, increased.

### 2.3 Tuberculous Endocarditis

Tuberculous endocarditis as a complication of TB is extremely rare, and just a few case reports are described in the literature, and there is a notable absence of large-scale studies [10, 34, 35]. *M. tuberculosis* reaches the endocardium through hematogenous spread, directly attaching and colonizing the valve tissue. This process activates specific molecules such as fibrinogen and other surface proteins (clumping factors, coagulase), leading to prolonged bacterial adhesion, the formation of new fibrin and platelets, resulting in vegetations and the formation of granulomas. These vegetations produce toxins that damage the valves, starting a pathological cycle—a broken valve further promotes the fibrin-platelet aggregation and activation of the extrinsic clotting pathway, which may contribute to cytokine storm. As the vegetations continue to grow, they stimulate a pro-inflammatory chemokine response and even more active TB endocarditis development, which may cause the formation of circulating immune complexes and potentially damage other organs such as the kidneys, eyes, and skin. This severe and disseminated TB form is called miliary TB [36, 37]. Moreover, due to bacteriemia, there is an increased risk of abscess formation [38].

TB endocarditis manifests with non-specific systemic symptoms such as fever, weight loss, and heart failure if there is significant valve damage. Cough and hemoptysis, which are the main symptoms of pulmonary TB, are not common [38]. Most commonly, vegetations form in the aortic or mitral valve, destroying the heart’s leaflets and causing significant cardiac damage, leading to myocardial infarction or acute coronary syndrome. Less frequently, the tricuspid valve is affected, leading to an embolization to the lungs [36]. Clinically, valve damage may present as a new-onset cardiac murmur [36, 39].

The 2023 European Society of Cardiology (ESC) Guidelines for endocarditis recommend transthoracic echocardiography (TTE) and transesophageal echocardiography (TOE) as the first-line imaging tests. TTE is always performed due to its good availability and specificity, despite its low sensitivity (IB class recommendation). TOE is recommended for all patients with clinical suspicion of endocarditis, even if TTE is positive, because it more precisely evaluates valve damage, small vegetations, perivalvular complications, and endocarditis associated with a prosthetic valve or cardiac implantable electronic device [39].

TB endocarditis may be diagnosed based on modified Duke criteria for infective endocarditis, which include major criteria, such as positive blood culture and specific imaging findings of infective endocarditis (IE), as well as minor criteria, including various clinical symptoms (Table 5) [39]. *M. tuberculosis* grows slowly in blood culture, or even the test may often be negative; therefore, imaging tests and various minor criteria are commonly used to confirm the diagnosis of TB endocarditis [36, 37].

**Table 5.** **The 2023 ESC modified diagnostic criteria of infective endocarditis**.

Major criteria			
1. Blood cultures positive for IE			
Microorganisms consistent with IE from continuously positive blood cultures:			
	a. ≥2 positive blood cultures of blood samples drawn >12 h apart.		
	b. All of 3 or a majority of ≥4 separate cultures of blood (with first and last samples drawn ≥1 h apart).		
2. Imaging positive for IE			
Valvular, perivalvular/periprosthetic, and foreign material anatomic and metabolic lesions characteristic of IE detected by any of the following imaging techniques:			
	a. Echocardiography (TTE and TOE).		
	b. Cardiac CT.		
	c. [^18^F]-FDG-PET/CT(A).		
	d. WBC SPECT/CT.		
Minor criteria			
1. Predisposing conditions (i.e., predisposing heart condition at high or intermediate risk of IE or PWIDs)			
2. Fever is defined as a temperature >38 °C			
3. Embolic vascular dissemination (including those asymptomatic, detected by imaging only):			
	a. Major systemic and pulmonary emboli/infarcts and abscesses.		
	b. Hematogenous osteoarticular septic complications (i.e., spondylodiscitis).		
	c. Mycotic aneurysms.		
	d. Intracranial ischemic/hemorrhagic lesions.		
	e. Conjunctival hemorrhages.		
	f. Janeway’s lessons.		
4. Immunological phenomena:			
	a. Glomerulonephritis.		
	b. Osler nodes and Roth spots.		
	c. Rheumatoid factor.		
5. Microbiological evidence:			
	a. Positive blood culture, but does not meet a major criterion as noted above.		
IE Classification (at admission and during follow-up)			
Definite:		Possible:	Rejected:
∙ 2 major criteria.		∙ 1 major criterion and 1 or 2 minor criteria.	∙ Does not meet criteria for definite or possible at admission with or without a firm alternative diagnosis.
∙ 1 major criterion and at least 3 minor criteria.		∙ 3–4 minor criteria.	
∙ 5 minor criteria.			

[^18^F]-FDG-PET/CT, 18F-fluorodeoxyglucose positron emission tomography; CT(A), computed tomography (angiography); IE, infective endocarditis; Ig, immunoglobulin; PWID, people who inject drugs; TOE, transesophageal echocardiography; TTE, transthoracic echocardiography; WBC SPECT/CT, white blood cell single photon emission tomography/computed tomography; ESC, European Society of Cardiology.

If the diagnosis of IE is unclear, the gold standard is histopathological examination, which can be performed by collecting damaged tissue or embolic fragments during valve surgery. During this examination, the most characteristic features of TB endocarditis are nodules on the valves, caseating granulomas, polypoidal tubercles, and thrombi containing *M. tuberculosis* bacilli [36, 39].

Treatment of TB endocarditis is similar to TB pericarditis and other TB-related cases, involving a combination of anti-TB drugs for six months. However, if the valves are completely damaged, surgical replacement may be necessary [10, 36]. Importantly, all *Mycobacterium* species can exhibit resistance to antibacterial therapy, which is associated with a higher relapse rate of IE. Therefore, appropriate treatment, active multidisciplinary (cardiologists, infectious disease specialists, cardiac surgeons, general practitioners) follow-up, and post-treatment prophylaxis are essential [39].

### 2.4 Tuberculous Aortitis

TB aortitis is a rare type of cardiac involvement in tuberculosis, but it has potentially good outcomes if diagnosed in time. A single-center study in Paris investigated 108 cases of tuberculosis and estimated that 3 patients (2.8%) were complicated by aortitis. Notably, all of them showed clinical improvement after treatment [40, 41].

*M. tuberculosis* can infect the aorta through lymphogenic or paravertebral abscess, hematogenous spread, and additionally, it can directly contaminate atherosclerotic plaques in the aorta [28, 42]. The TB infection in the aorta often leads to dilatation, which causes a pseudoaneurysm (mycotic aneurysm), mostly in the thoracic or abdominal part of the aorta, and less frequently it presents as aortic stenosis [40, 42]. Predominantly, pseudoaneurysms are asymptomatic and are often detected accidentally. In addition, it may present with chest pain, compression, weight loss, fever, pain in various parts of the body, or other nonspecific symptoms, which complicate the diagnostic process [40, 43, 44]. Moreover, if the aorta is stenotic, it may cause claudication, dyspnea, or syncope and may lead to heart failure [40, 45].

Mycotic aneurysms are most accurately diagnosed through imaging techniques, as there are no specific laboratory tests, and blood culture may not always be positive in cases of TB. The CT angiography (CTA) is considered the gold standard for early diagnosis, planning surgical or endovascular treatment, and assessing the risk of complications. Other imaging tests, such as CT and magnetic resonance angiography (MRA), may also be used, but are not as fast and accurate as CTA [43].

The treatment of TB aortitis/pseudoaneurysm is variable and includes anti-TB therapy for small and asymptomatic aneurysms, as well as open or endovascular procedures for advanced aneurysms, or a combination of medical and surgical treatments [43].

Although usually there are good outcomes, early diagnosis and a multidisciplinary approach are essential to prevent patients from sepsis, aortic rupture, or dissection resulting in death [40, 43].

### 2.5 Cardiovascular Disease

#### 2.5.1 Mechanism

The link between infection and cardiovascular disease (CVD), which are two distinct health conditions, has been studied for many years, and it was found that both acute and chronic TB infections have a significant influence on coronary arteries and increase overall cardiovascular events. Literature suggests that coronary arteries may be strongly associated with chronic endothelial inflammation caused by TB infection, when increased levels of adenosine deaminase in the plasma stimulate neutrophils to secrete free oxygen radicals, contributing to vascular damage [46]. Moreover, a systematic review and meta-analysis found that statin therapy may help to reduce active TB infection through various mechanisms [47, 48]. The primary mechanism is the inhibition of cholesterol synthesis, as cholesterol is essential for *M. tuberculosis* entry into host cells, such as macrophages [47]. Additionally, some *in vitro* studies suggest that fluvastatin may enhance the immune response in cases of TB infection by increasing cytokine release from Th1 cells and activating caspase 1 [48].

#### 2.5.2 Epidemiology

Acute TB infection considerably doubles the risk of subsequent acute MI for 1 year, and latent TB infection increases all types of CVD risk by 8% [49, 50, 51]. Additionally, individuals with TB have a 51% increased risk of major adverse cardiovascular events, such as cardiovascular mortality, acute MI, unstable angina, and nonfatal stroke, compared to non-TB individuals. This elevated risk is attributable to increased inflammation, heat shock protein-mediated autoimmunity, and *M. tuberculosis* contamination, which all lead to increased morbidity and mortality [6, 52]. Latent TB infection is strongly associated with and independently contributes to an increased risk of chronic obstructive coronary artery disease [46]. This link is highlighted in one study, which showed that 9% of all patients with latent TB had obstructed coronary arteries, compared to 3% in TB-negative people [53].

#### 2.5.3 Special Scenarios

Moreover, arterial hypertension, the primary determinant of CVD, is also associated with latent TB. TB-positive patients have a higher risk of developing hypertension, which is less controllable than in TB-negative individuals, particularly in those without other CVD risk factors, such as a high body mass index, hyperglycemia, or smoking [54, 55].

Although TB can be treated with generally good outcomes, TB survivors exhibited a 21% higher risk of developing ischemic heart disease and a 48% greater risk of myocardial infarction compared to matched controls, and the reason is unclear [56].

### 2.6 Cardiac Tuberculoma 

Tuberculomas are encapsulated granulomatous lesions caused by *M. tuberculosis*. They most commonly develop in the lungs, but may occur in other organs. Cardiac tuberculomas are extremely rare and typically are located in the right heart, particularly on the free right atrial wall, and this tendency is explained by the fact that mediastinal lymph nodes and lymphatic vessels on the right side are drained directly into the right subclavian vein and right atrium [4, 57]. However, there are some case reports in the literature describing tuberculomas in the left heart [58, 59].

Moreover, depending on their localization, tuberculomas can be classified as intracardiac and intracavitary. Intracardiac tuberculomas are less common and are located in the heart tissue, most commonly in the myocardium, whereas intracavitary tuberculomas are found in the heart chambers, usually in the right heart, as previously mentioned [4]. Clinically, tuberculomas may present with general symptoms (fever, night sweats, weight loss, weakness), can be asymptomatic or even present as sudden cardiac death [60, 61, 62]. Intracavitary tuberculomas may obstruct the right ventricular outflow tract, the superior vena cava, or even the coronary artery ejection tracts. This may have an impact on cardiac function and may lead to ventricular dysfunction, ventricular rupture, or different arrhythmias [62].

Intracardiac tuberculomas can be detected using imaging tests, such as echocardiography, FDG-PET/CT, and cardiovascular magnetic resonance imaging, which reveals isointense central caseation, a hypointense fibrous capsule, and a hyperintense line of inflammatory cellular infiltration. However, only histopathological examination and mycobacterial culture can confirm the diagnosis of TB, as it carries a substantial risk of being misdiagnosed as a cardiac tumor, particularly in immunosuppressed individuals [59, 62].

Cases of tuberculomas require special attention and a multidisciplinary approach by healthcare professionals to find the most appropriate individual treatment. There are no specific guidelines for the duration of anti-TB therapy and when surgery should be indicated. However, surgery is recommended in cases of uncertain diagnosis, hemodynamically unstable patients with significant obstruction, or when there is an inadequate response to medical treatment [4, 59].

### 2.7 Anti-TB Drug Cardiotoxicity

Although *M. tuberculosis* affects the heart function differently, anti-TB treatment carries its own cardiotoxic risks, especially, speaking about treatment used for multi-drug resistant TB (MDR-TB) [4, 63, 64]. MDR-TB is a form of TB that is resistant to rifampicin and isoniazid, the most commonly used drugs for TB treatment. Drugs for MDR-TB treatment according to the WHO 2022 update are described in Table 6 [64]. Bedaquiline is strongly associated with prolonging the QTc interval due to inhibition of the potassium channel *hERG* (human Ether-à-go-Related Gene, which is encoded by the *KCNH2*). This leads to delayed ventricular repolarization and the left ventricle becomes more sensitive to premature electrical impulses, which increases the risk of polymorphic ventricular tachycardia, also known as *Torsades de pointes* [63, 65, 66].

**Table 6.** **Treatment: Drug-resistant tuberculosis treatment**.

Group and steps	Medicine
Group A:	Levofloxacin or moxifloxacin
Include all three medicines	Bedaquiline
	Linezolid
Group B:	Clofazimine
Add one or both medicines	Cycloserine or terizidone
Group C:	Ethambutol
Add to complete the regimen, and when medicines from Groups A and B cannot be used	Delamanid
	Pyrazinamide
	Imipenem-cilastatin or meropenem
	Amikacin or streptomycin
	Ethionamide or prothionamide
	P-aminosalicylic acid

Other anti-TB drugs, such as fluoroquinolones (levofloxacin, moxifloxacin) and clofazimine, have similar effects and should not be combined [63, 65, 66]. Furthermore, the literature reports several cases of drug-induced cardiomyopathy from anti-TB treatment, including two patients who developed dilated cardiomyopathy [67]. Another report describes isoniazid-induced drug rash accompanied by eosinophilic myocarditis and fever [68]. Although drug-induced cardiac lesions are extremely rare, these cases highlight the importance of careful monitoring in patients receiving anti-TB therapy.

## 3. Conclusions

Cardiac complications in tuberculosis are infrequent, and the most common cardiovascular involvement in tuberculosis is pericarditis. Moreover, it has a significant effect on increasing the risk of cardiovascular diseases, such as myocardial infarction or coronary artery obstruction. All in all, it is essential to make an accurate diagnosis as early as possible and initiate adequate treatment to prevent patients from life-threatening outcomes, such as sudden cardiac death, sepsis, or aortic rupture. A multidisciplinary healthcare approach is essential for optimizing patient management and improving prognosis.

## References

[1] Pai M, Behr MA, Dowdy D, Dheda K, Divangahi M, Boehme CC (2016). Tuberculosis. * Nature Reviews. Disease Primers*.

[2] (2024). Global tuberculosis report 2024.

[3] Duarte R, Lönnroth K, Carvalho C, Lima F, Carvalho ACC, Muñoz-Torrico M (2018). Tuberculosis, social determinants and co-morbidities (including HIV). *Pulmonology*.

[4] Marcu DTM, Adam CA, Mitu F, Cumpat C, Aursulesei Onofrei V, Zabara ML (2023). Cardiovascular Involvement in Tuberculosis: From Pathophysiology to Diagnosis and Complications-A Narrative Review. *Diagnostics*.

[5] Imazio M, Gaita F, LeWinter M (2015). Evaluation and Treatment of Pericarditis: A Systematic Review. *JAMA*.

[6] Shabil M, Bushi G, Beig MA, Rais MA, Ahmed M, Padhi BK (2023). Cardiovascular Manifestation in Tuberculosis Cases: A Systematic Review and Meta-Analysis. *Current Problems in Cardiology*.

[7] Adler Y, Charron P (2015). The 2015 ESC Guidelines on the diagnosis and management of pericardial diseases. *European Heart Journal*.

[8] Cremer PC, Klein AL, Imazio M (2024). Diagnosis, Risk Stratification, and Treatment of Pericarditis: A Review. *JAMA*.

[9] Isiguzo G, Du Bruyn E, Howlett P, Ntsekhe M (2020). Diagnosis and Management of Tuberculous Pericarditis: What Is New?. *Current Cardiology Reports*.

[10] López-López JP, Posada-Martínez EL, Saldarriaga C, Wyss F, Ponte-Negretti CI, Alexander B (2021). Tuberculosis and the Heart. *Journal of the American Heart Association*.

[11] Howlett P, Du Bruyn E, Morrison H, Godsent IC, Wilkinson KA, Ntsekhe M (2020). The immunopathogenesis of tuberculous pericarditis. *Microbes and Infection*.

[12] Westin O, Qayyum AA (2019). Recurrent Episodes of Pericardial Effusion as Isolated Manifestation of Tuberculosis: Case Report. *Current Medical Imaging Reviews*.

[13] Rustad AM, Hughes ZH, Osborn RL, Bhasin A (2022). Non-pulmonary Disseminated Tuberculosis Complicated by Constrictive Pericarditis and Cutaneous Gumma. *Journal of General Internal Medicine*.

[14] Khatun N, Akivis Y, Ji B, Chandrakumar HP, Bukharovich I, John S (2023). Tuberculous Pericarditis Presenting as Cardiac Tamponade: Role of Echocardiography. *Journal of Medical Cases*.

[15] Ntsekhe M, Mayosi BM (2013). Tuberculous pericarditis with and without HIV. *Heart Failure Reviews*.

[16] Jung IY, Song YG, Choi JY, Kim MH, Jeong WY, Oh DH (2016). Predictive factors for unfavorable outcomes of tuberculous pericarditis in human immunodeficiency virus-uninfected patients in an intermediate tuberculosis burden country. *BMC Infectious Diseases*.

[17] Pérez-Casares A, Cesar S, Brunet-Garcia L, Sanchez-de-Toledo J (2017). Echocardiographic Evaluation of Pericardial Effusion and Cardiac Tamponade. *Frontiers in Pediatrics*.

[18] Brandt RR, Oh JK (2017). Constrictive pericarditis: role of echocardiography and magnetic resonance imaging. https://www.escardio.org/Journals/E-Journal-of-Cardiology-Practice/Volume-15/Constrictive-pericarditis-role-of-echocardiography-and-magnetic-resonance-imaging.

[19] Dybowska M, Błasińska K, Gątarek J, Klatt M, Augustynowicz-Kopeć E, Tomkowski W (2022). Tuberculous Pericarditis-Own Experiences and Recent Recommendations. *Diagnostics*.

[20] Gumedze F, Pandie S, Nachenga JB, Kerbelker Z, Francis V, Thabane L (2023). The impact of HIV co-infection on presentation and outcome in adults with tuberculous pericarditis: Findings from the IMPI trial. *SAMJ: South African Medical Journal*.

[21] Matthews K, Ntsekhe M, Syed F, Scriba T, Russell J, Tibazarwa K (2012). HIV-1 infection alters CD4+ memory T-cell phenotype at the site of disease in extrapulmonary tuberculosis. *European Journal of Immunology*.

[22] Kim MS, Kim EK, Choi JY, Oh JK, Chang SA (2019). Clinical Utility of [18F]FDG-PET/CT in Pericardial Disease. *Current Cardiology Reports*.

[23] Chau E, Sarkarati M, Spellberg B (2019). Adenosine Deaminase Diagnostic Testing in Pericardial Fluid. *JAMA*.

[24] Shenje J, Ifeoma Adimora-Nweke F, Ross IL, Ntsekhe M, Wiesner L, Deffur A (2015). Poor Penetration of Antibiotics Into Pericardium in Pericardial Tuberculosis. *eBioMedicine*.

[25] Mayosi BM, Ntsekhe M, Bosch J, Pandie S, Jung H, Gumedze F (2014). Prednisolone and Mycobacterium indicus pranii in tuberculous pericarditis. *The New England Journal of Medicine*.

[26] Wiysonge CS, Ntsekhe M, Thabane L, Volmink J, Majombozi D, Gumedze F (2017). Interventions for treating tuberculous pericarditis. *The Cochrane Database of Systematic Reviews*.

[27] Kumar S, Bhutani N, Kataria SP, Sen R (2018). Tuberculous myocarditis on autopsy: a rare underdiagnosed entity. *Cardiovascular Pathology*.

[28] Mutyaba AK, Ntsekhe M (2017). Tuberculosis and the Heart. *Cardiology Clinics*.

[29] Michira BN, Alkizim FO, Matheka DM (2015). Patterns and clinical manifestations of tuberculous myocarditis: a systematic review of cases. *The Pan African Medical Journal*.

[30] du Toit-Prinsloo L, Saayman G (2016). “Death at the wheel” due to tuberculosis of the myocardium: a case report. *Cardiovascular Pathology*.

[31] Cowley A, Dobson L, Kurian J, Saunderson C (2017). Acute myocarditis secondary to cardiac tuberculosis: a case report. *Echo Research and Practice*.

[32] Mohan A, Thachil A, Sundar G, Sastry BKS, Hasan A, Sridevi C (2015). Ventricular tachycardia and tuberculous lymphadenopathy: sign of myocardial tuberculosis?. *Journal of the American College of Cardiology*.

[33] Chabior A, Tymińska A, Pawlak A, Giordani A, Caforio A, Grabowski M (2024). Advances in myocarditis management in the light of the latest research and recent guidelines of the European Society of Cardiology. *Cardiology Journal*.

[34] Saboe A, Sakasasmita S, Hartantri Y, Maryani E, Hadar AK, Sudjud RW (2021). A case of endocarditis and spondylodiscitis associated with Mycobacterium tuberculosis. *IDCases*.

[35] El Hangouche AJ, Oukerraj L (2017). Mycobacterium tuberculosis endocarditis in native valves. *The Pan African Medical Journal*.

[36] Dawi J, Affa S, Chorbajian A, Misakyan Y, Mohan AS, Norris B (2024). Mycobacterial Endocarditis, A Rare Form of Infective Endocarditis. *Discovery Medicine*.

[37] Vohra S, Dhaliwal HS (2024). Miliary Tuberculosis. http://www.ncbi.nlm.nih.gov/books/NBK562300/.

[38] Liu A, Nicol E, Hu Y, Coates A (2013). Tuberculous endocarditis. *International Journal of Cardiology*.

[39] Delgado V, Ajmone Marsan N, de Waha S, Bonaros N, Brida M, Burri H (2023). 2023 ESC Guidelines for the management of endocarditis. *European Heart Journal*.

[40] Delaval L, Goulenok T, Achouh P, Saadoun D, Gaudric J, Pellenc Q (2017). New insights on tuberculous aortitis. *Journal of Vascular Surgery*.

[41] Pathirana U, Kularatne S, Karunaratne S, Ranasinghe G, Fernando J (2015). Ascending aortic aneurysm caused by Mycobacterium tuberculosis. *BMC Research Notes*.

[42] Manika K, Efthymiou C, Damianidis G, Zioga E, Papadaki E, Lagoudi K (2017). Miliary tuberculosis in a patient with tuberculous mycotic aneurysm of the abdominal aorta: Case report and review of the literature. *Respiratory Medicine Case Reports*.

[43] Abdelazeem B, Kambalapalli S, Lahmar A, Yousaf A, Kusz H (2022). Infectious Aortitis: Case Report and Literature Review. *Cureus*.

[44] Mazzolai L, Teixido-Tura G, Lanzi S, Boc V, Bossone E, Brodmann M (2024). 2024 ESC Guidelines for the management of peripheral arterial and aortic diseases. *European Heart Journal*.

[45] Pujari SH, Agasthi P (2023). Aortic Stenosis. http://www.ncbi.nlm.nih.gov/books/NBK557628/.

[46] Murillo Moreno MA, López Gutiérrez LV, Vinck EE, Roncancio Villamil G, Gallego Muñoz C, Saldarriaga Giraldo CI (2024). Coronary heart disease and tuberculosis: an unnoticed syndemia. Review of literature and management proposal. *Archivos Peruanos De Cardiologia Y Cirugia Cardiovascular*.

[47] Duan H, Liu T, Zhang X, Yu A, Cao Y (2020). Statin use and risk of tuberculosis: a systemic review of observational studies. *International Journal of Infectious Diseases*.

[48] Li X, Sheng L, Lou L (2020). Statin Use May Be Associated with Reduced Active Tuberculosis Infection: A Meta-Analysis of Observational Studies. *Frontiers in Medicine*.

[49] Hossain MB, Johnston JC, Cook VJ, Sadatsafavi M, Wong H, Romanowski K (2023). Role of latent tuberculosis infection on elevated risk of cardiovascular disease: a population-based cohort study of immigrants in British Columbia, Canada, 1985-2019. *Epidemiology and Infection*.

[50] Huaman MA, Henson D, Ticona E, Sterling TR, Garvy BA (2015). Tuberculosis and Cardiovascular Disease: Linking the Epidemics. *Tropical Diseases, Travel Medicine and Vaccines*.

[51] Huaman MA, Kryscio RJ, Fichtenbaum CJ, Henson D, Salt E, Sterling TR (2017). Tuberculosis and risk of acute myocardial infarction: a propensity score-matched analysis. *Epidemiology and Infection*.

[52] Basham CA, Smith SJ, Romanowski K, Johnston JC (2020). Cardiovascular morbidity and mortality among persons diagnosed with tuberculosis: A systematic review and meta-analysis. *PLoS ONE*.

[53] Huaman MA, De Cecco CN, Bittencourt MS, Ticona E, Kityo C, Ballena I (2021). Latent Tuberculosis Infection and Subclinical Coronary Atherosclerosis in Peru and Uganda. *Clinical Infectious Diseases*.

[54] Salindri AD, Auld SC, Gujral UP, Urbina EM, Andrews JR, Huaman MA (2024). Tuberculosis infection and hypertension: prevalence estimates from the US National Health and Nutrition Examination Survey. *BMJ Open*.

[55] Mandieka E, Saleh D, Chokshi AK, Rivera AS, Feinstein MJ (2020). Latent Tuberculosis Infection and Elevated Incidence of Hypertension. *Journal of the American Heart Association*.

[56] Lee HR, Yoo JE, Choi H, Han K, Lim YH, Lee H (2023). Tuberculosis and the Risk of Ischemic Heart Disease: A Nationwide Cohort Study. *Clinical Infectious Diseases*.

[57] Sabzi F, Faraji R, Khoshnood S (2024). Right atrial tuberculoma enclosed by thrombus. *Global Cardiology Science & Practice*.

[58] Kemaloğlu Öz T, Elsayed M, Nanda NC, Kalenderoğlu K, Akyüz Ş, Atasoy I (2016). Incremental value of live/real time three-dimensional transesophageal echocardiography over the two-dimensional technique in the assessment of a tuberculoma involving the left atrium and appendage. *Echocardiography*.

[59] Rangaswamy VV, Saggu DK, Penmetcha K, Yalagudri S, Calambur N (2020). Mitral valve tuberculoma: Role of sequential multimodality imaging of an unusual intracardiac mass. *Echocardiography*.

[60] Lema AS (2023). Cardiac Tuberculoma Presenting as Sudden Cardiac Death in an Immunocompetent Young Man: A Case Report and Literature Review. *Case Reports in Cardiology*.

[61] Säll O, Cha SO, Holmberg H (2016). Diagnostic challenges in a patient with myocardial tuberculoma: A case report. *International Journal of Surgery Case Reports*.

[62] Das KM, Mansoori TA, Alattar YH, Gorkom KV, Shamisi A, Melethil AP (2022). Tuberculosis of the Heart: A Diagnostic Challenge. *Tomography*.

[63] Polak S, Romero K, Berg A, Patel N, Jamei M, Hermann D (2018). Quantitative approach for cardiac risk assessment and interpretation in tuberculosis drug development. *Journal of Pharmacokinetics and Pharmacodynamics*.

[64] World Health Organization (2022). *WHO Operational Handbook on Tuberculosis. Module 4: Treatment - Drug-Resistant Tuberculosis Treatment, 2022 Update. 1st edn*.

[65] Patel H, Pawara R, Pawara K, Ahmed F, Shirkhedkar A, Surana S (2019). A structural insight of bedaquiline for the cardiotoxicity and hepatotoxicity. *Tuberculosis*.

[66] Jin Y, Benkeser D, Kipiani M, Maranchick NF, Mikiashvili L, Barbakadze K (2023). The effect of anti-tuberculosis drug pharmacokinetics on QTc prolongation. *International Journal of Antimicrobial Agents*.

[67] Ray A, Nangia V, Chatterji RS, Dalal N, Ray RS (2017). Recurrent heart failure in pulmonary tuberculosis patients on antitubercular therapy: A case of protector turning predator. *Egyptian Journal of Bronchology*.

[68] Zhang SN, He QX, Yang NB, Ni SL, Lu MQ (2015). Isoniazid-induced Drug Rash with Eosinophilia and Systemic Symptoms (DRESS) Syndrome Presenting as Acute Eosinophilic Myocarditis. *Internal Medicine*.

